# Supplementary material for: Dysregulation of alternative splicing patterns in the ovaries of reproductively aged mice
Source: Reproduction. 2026 Feb 2;171(3):xaag019. doi: 10.1093/reprod/xaag019 (PMC13046076; doi:10.1093/reprod/xaag019)
Supplement: xaag019_Supplementary_Data [file xaag019_supplementary_data.docx]

**Supplemental Figures**


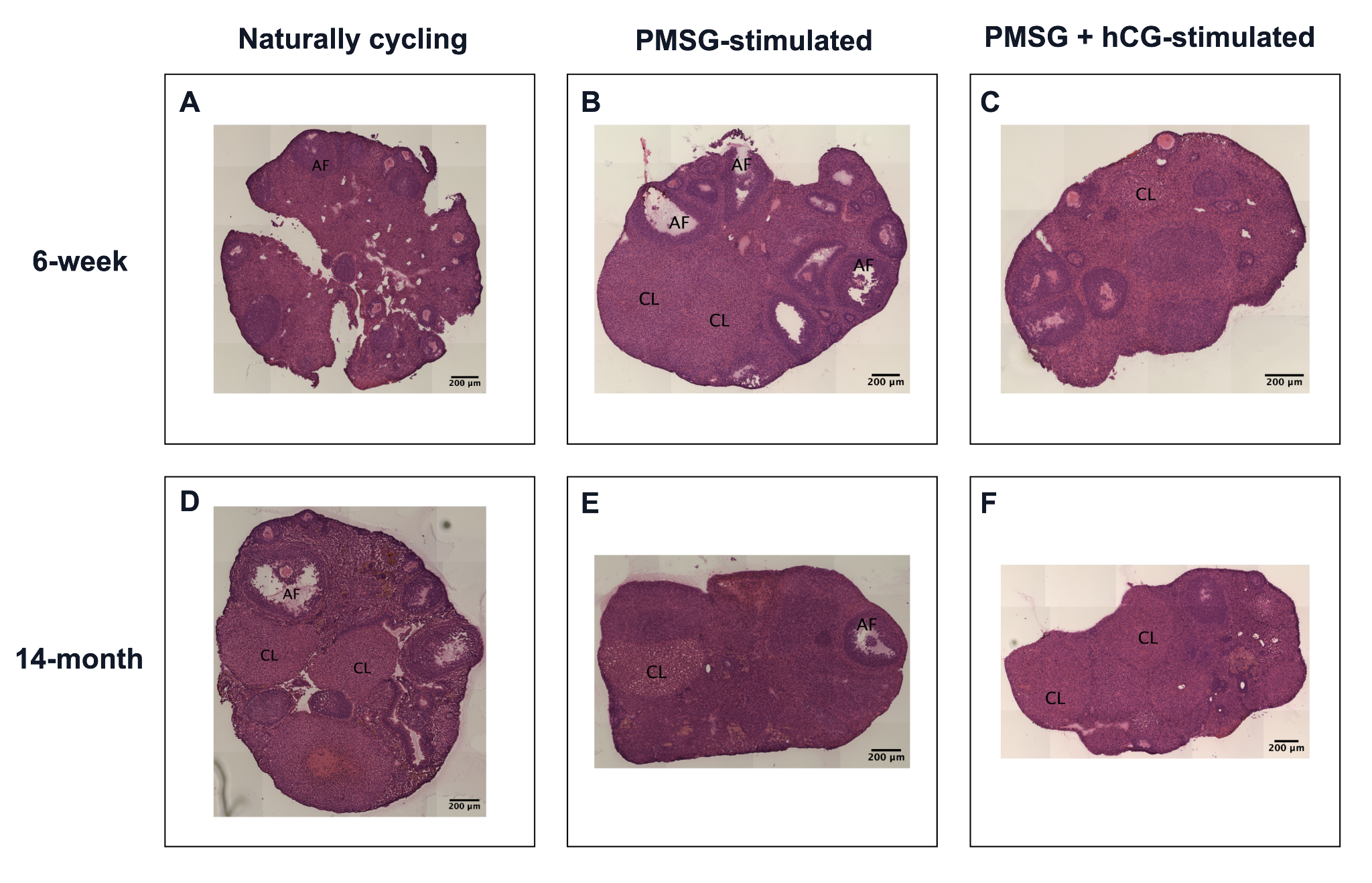


**Supplementary Figure S1. Hematoxylin & eosin staining of 6-week and 14-month mouse ovaries from naturally cycling and gonadotropin stimulated females.** H&E-stained ovaries from 6-week-old mice are shown for (A) naturally cycling, (B) PMSG-stimulated, and (C) PMSG+hCG-stimulated. H&E-stained ovaries from 14-month-old mice are shown for (D) naturally cycling, (E) PMSG-stimulated, and (F) PMSG+hCG-stimulated. Corpora lutea (CL) and antral follicles (AF) are indicated.


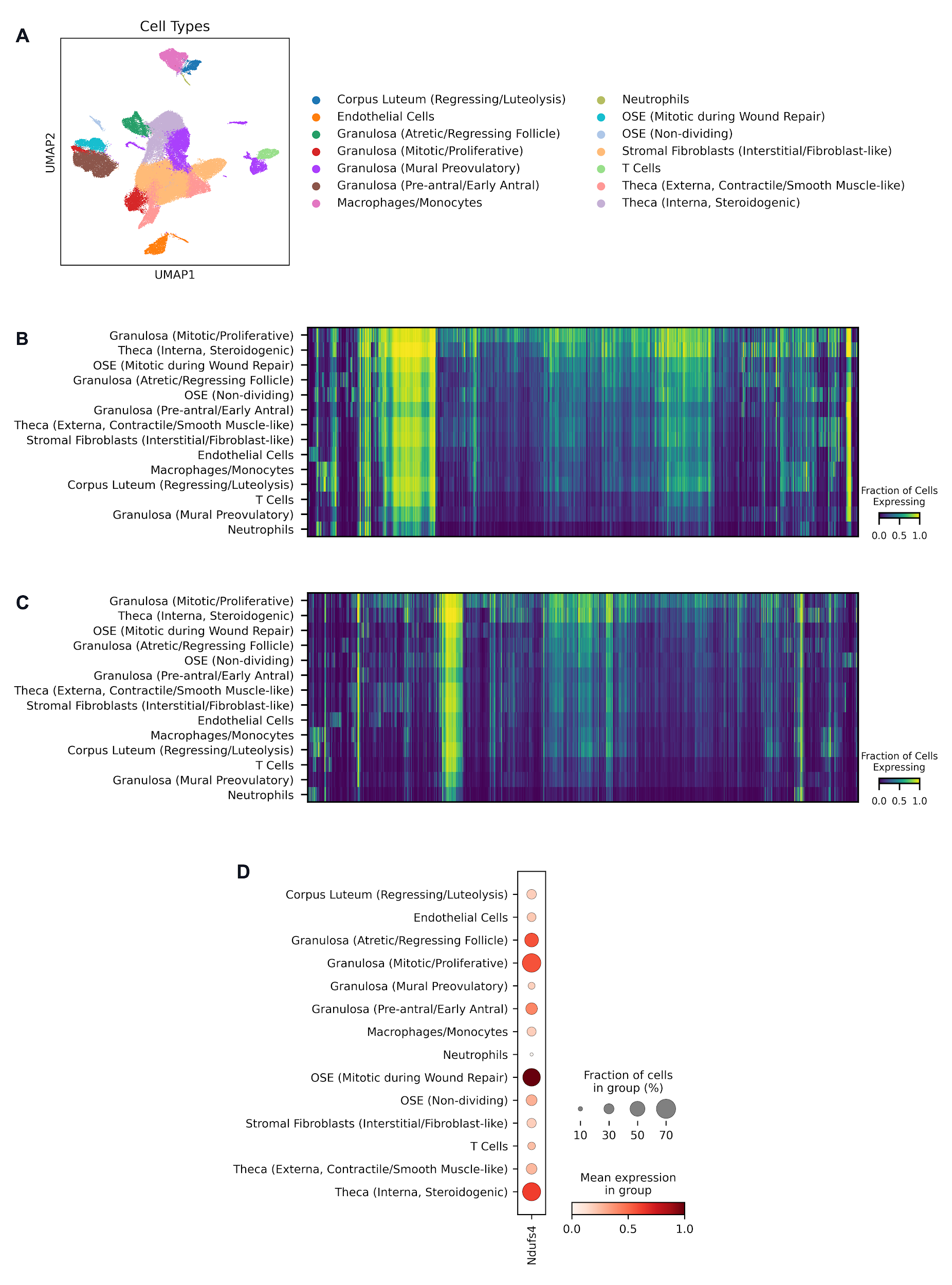


**Supplementary Figure S2. Cell-type mapping of age-associated splice isoforms in the ovary.** (A) UMAP plot of cell populations from 3-month-old C57BL/6J and C57BL/6Ly5.1 mouse ovaries. Clustering analysis revealed 14 distinct ovarian cell populations. (B - C) Heatmaps display the fraction of cells with detectable gene expression (0 - 1) within each scRNA-seq-defined ovarian cell type (rows) for each gene (columns). Columns correspond to genes (gene-level, not isoform-level) that harbor ≥ 1 transcript with significant age-associated alternative splicing in the corresponding long-read comparison (PMSG in B; PMSG + hCG in C). The contiguous high-intensity (yellow) band represents genes detected in most cells across essentially all cell types (ubiquitous/housekeeping-like genes); Genes driving the ubiquitous high‑fraction band across cell types (yellow stripe) and their Reactome enrichment results are provided in Supplementary Table S6.


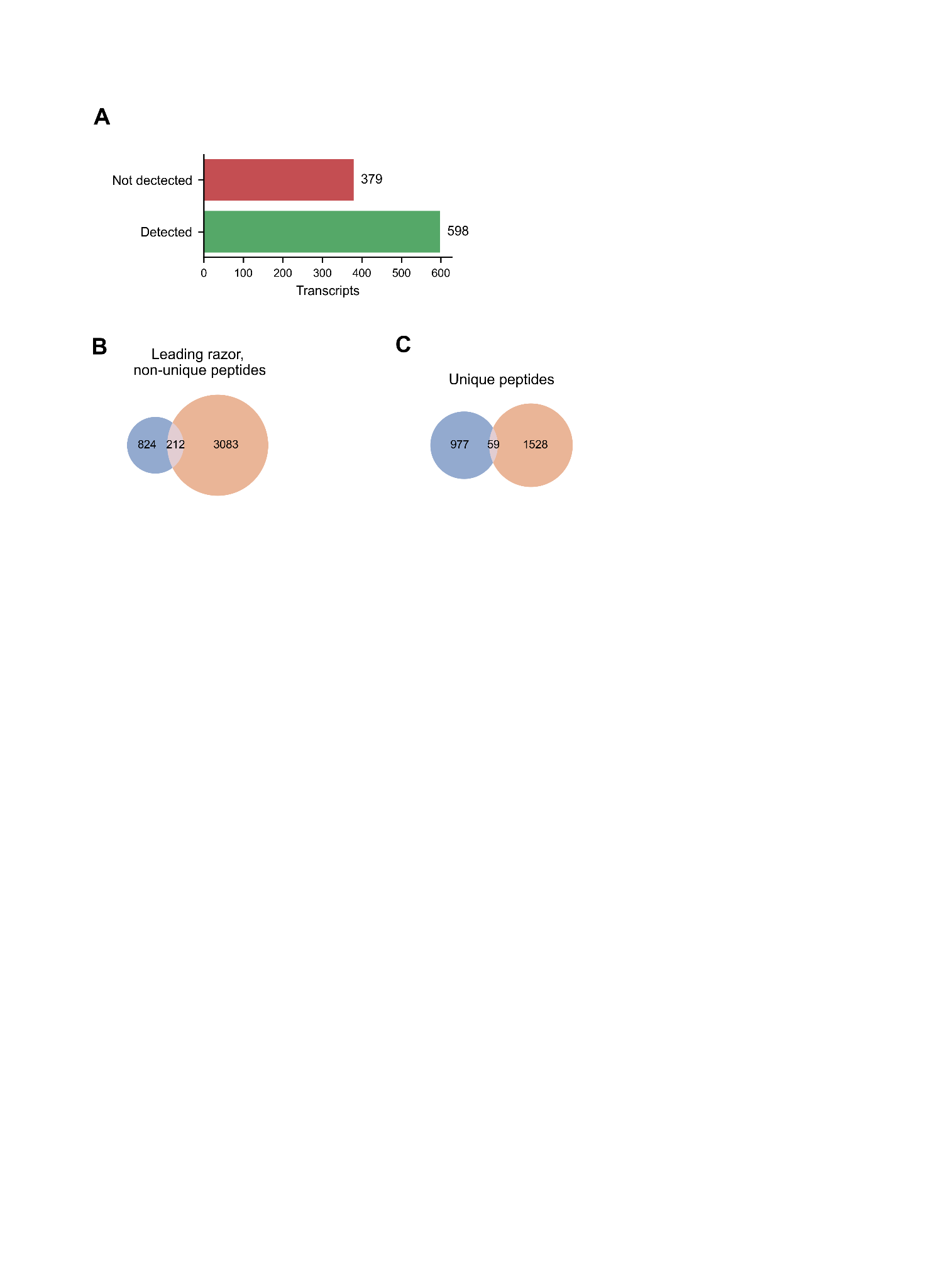


**Supplementary Figure S3. Proteomics overlap with PFAM disrupted transcripts.** (A) Red and green horizontal bars show how many of the PFAM disrupted transcripts were detected in all-protein groups (B) Overlap between transcripts with a PFAM domain annotation (blue) compared to a *more permissive* MS set (orange). This panel shows how relaxing MS filters changes the overlap with PFAM‑annotated transcripts. (C) Same PFAM set (blue) and a *strict* mass‑spectrometry (MS) set (orange). The intersection of both venn diagrams shows transcripts supported by both approaches. See Methods.


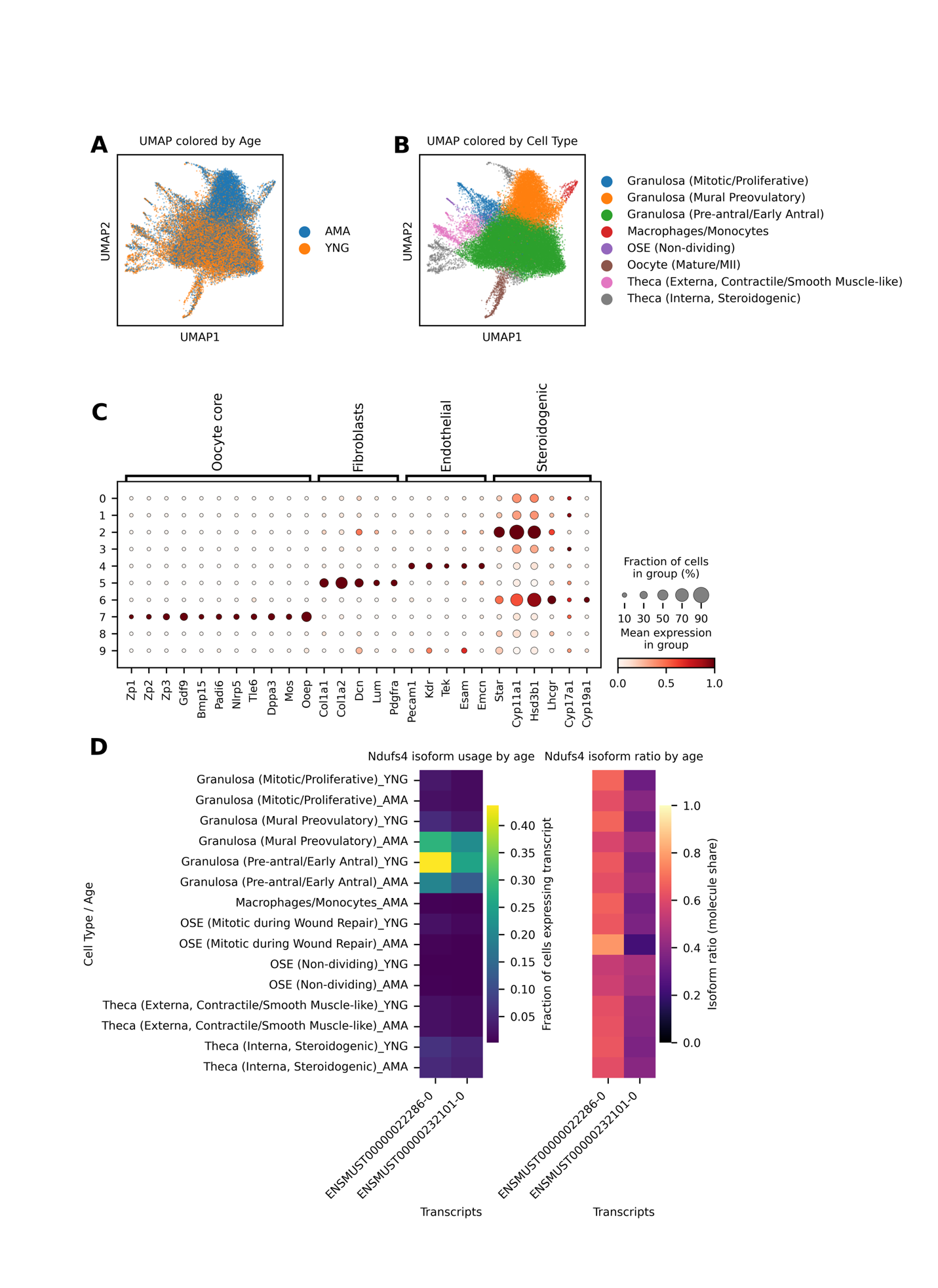


**Supplementary Figure S4. Single-cell RNA-sequencing of young (YNG) and aged (AMA) mouse ovaries.** UMAP embeddings from ovaries of young (6-week) and aged (14-month) C57BL/6 mice colored by (A) age and by (B) annotated cell types. (C) Canonical-marker dot plot summarizing cluster identities. (D) *Ndufs4* isoform usage and within-promoter isoform ratios across cell types and ages.

Supplementary Table S1: Marker genes to annotate cell clusters

| **Cell types** | **Gene markers** |
| --- | --- |
| Corpus Luteum (Regressing/Luteolysis) | Cdkn1a, Fas, Il1b, Mmp9 [(2–5)](https://paperpile.com/c/RdymOk/qPVO+1JAV+C49g+uWtO) |
| Endothelial Cells | Emcn, Esam, Kdr, Pecam1, Tek [(6–10)](https://paperpile.com/c/RdymOk/SD2B+iO0O+Sj61+CuaB+vVxZ) |
| Granulosa (Atretic/Regressing Follicle) | Gadd45a, Ghr, Mitf, Nupr1, Pik3ip1, Vat1 [(11–16)](https://paperpile.com/c/RdymOk/lrAG+9g8v+VnKi+bm6N+CCq3+QYgK) |
| Granulosa (Mitotic/Proliferative) | Mki67, Top2a [(17,18)](https://paperpile.com/c/RdymOk/pCwm+RO2W) |
| Granulosa (Mural Preovulatory) | Adamts1, Areg, Btc, Edn2, Egr1, Ereg, Klf4, Nr4a1, Pgr, Ptgs2, Runx2, Cyp11a1, Lhcgr, Star [(5,19–27)](https://paperpile.com/c/RdymOk/B1y6+bl8J+uWtO+bl2l+mPtP+aHAN+G93D+t7ca+9ejV+uthA) |
| Granulosa (Pre-antral/Early Antral) | Amh, Esr2, Fshr, Fst, Gas6, Inha, Inhbb, Kctd14, Kitl [(5,14,24,25,28–31)](https://paperpile.com/c/RdymOk/uWtO+bm6N+t7ca+gUIu+4V5q+oLIF+G93D+7gU8) |
| Macrophages/Monocytes | Adgre1, Csf1r, Itgam, Lyz2 [(4)](https://paperpile.com/c/RdymOk/C49g) |
| Neutrophils | Ly6g, S100a8, S100a9 [(4)](https://paperpile.com/c/RdymOk/C49g) |
| OSE (Mitotic during Wound Repair) | Birc5, Racgap1, Tk1, Ube2c [(32–34)](https://paperpile.com/c/RdymOk/dTgH+i1nG+j7YY) |
| OSE (Non-dividing) | Epcam, Krt18, Krt19, Krt7, Krt8, Muc1 [(35,36)](https://paperpile.com/c/RdymOk/BXbq+2LIP) |
| Stromal Fibroblasts (Interstitial/Fibroblast-like) | Col1a1, Col1a2, Cxcl14, Dcn, Kcnk2, Lum, Pdgfra [(5,24,25)](https://paperpile.com/c/RdymOk/uWtO+G93D+t7ca) |
| T Cells | Cd3d, Cd4, Cd8a, Trac [(4,35,37)](https://paperpile.com/c/RdymOk/BXbq+C49g+mct3) |
| Theca (Externa, Contractile/Smooth Muscle-like) | Acta2, Mfap5, Myh11, Tagln [(5,24,38)](https://paperpile.com/c/RdymOk/uWtO+G93D+1n21) |
| Theca (Interna, Steroidogenic) | Cyp17a1, Hsd3b1, Insl3 [(5,39,40)](https://paperpile.com/c/RdymOk/uWtO+dxuf+xl3C) |

​​Citations for single-cell RNA-sequencing annotation markers.

1. [Winkler I, Tolkachov A, Lammers F, Lacour P, Daugelaite K, Schneider N, et al. The cycling and aging mouse female reproductive tract at single-cell resolution. Cell. 2024 Feb 15;187(4):981–98.e25.](http://paperpile.com/b/RdymOk/Ovd8)

2. [Jirawatnotai S, Moons DS, Stocco CO, Franks R, Hales DB, Gibori G, et al. The cyclin-dependent kinase inhibitors p27Kip1 and p21Cip1 cooperate to restrict proliferative life span in differentiating ovarian cells. J Biol Chem. 2003 May 9;278(19):17021–7.](http://paperpile.com/b/RdymOk/qPVO)

3. [Carambula SF, Pru JK, Lynch MP, Matikainen T, Gonçalves PBD, Flavell RA, et al. Prostaglandin F2alpha- and FAS-activating antibody-induced regression of the corpus luteum involves caspase-8 and is defective in caspase-3 deficient mice. Reprod Biol Endocrinol. 2003 Feb 11;1:15.](http://paperpile.com/b/RdymOk/1JAV)

4. [Lee SK, Kim CJ, Kim DJ, Kang JH. Immune cells in the female reproductive tract. Immune Netw. 2015 Feb;15(1):16–26.](http://paperpile.com/b/RdymOk/C49g)

5. [Wagner M, Yoshihara M, Douagi I, Damdimopoulos A, Panula S, Petropoulos S, et al. Single-cell analysis of human ovarian cortex identifies distinct cell populations but no oogonial stem cells. Nat Commun. 2020 Mar 2;11(1):1147.](http://paperpile.com/b/RdymOk/uWtO)

6. [Zahr A, Alcaide P, Yang J, Jones A, Gregory M, dela Paz NG, et al. Endomucin prevents leukocyte-endothelial cell adhesion and has a critical role under resting and inflammatory conditions. Nat Commun. 2016 Feb 2;7(1):10363.](http://paperpile.com/b/RdymOk/SD2B)

7. [Ishida T, Kundu RK, Yang E, Hirata KI, Ho YD, Quertermous T. Targeted disruption of endothelial cell-selective adhesion molecule inhibits angiogenic processes in vitro and in vivo. J Biol Chem. 2003 Sep 5;278(36):34598–604.](http://paperpile.com/b/RdymOk/iO0O)

8. [Greenaway J, Connor K, Pedersen HG, Coomber BL, LaMarre J, Petrik J. Vascular endothelial growth factor and its receptor, Flk-1/KDR, are cytoprotective in the extravascular compartment of the ovarian follicle. Endocrinology. 2004 Jun;145(6):2896–905.](http://paperpile.com/b/RdymOk/Sj61)

9. [DeLisser HM, Christofidou-Solomidou M, Strieter RM, Burdick MD, Robinson CS, Wexler RS, et al. Involvement of endothelial PECAM-1/CD31 in angiogenesis. Am J Pathol. 1997 Sep;151(3):671–7.](http://paperpile.com/b/RdymOk/CuaB)

10. [Hata K, Udagawa J, Fujiwaki R, Nakayama K, Otani H, Miyazaki K. Expression of angiopoietin-1, angiopoietin-2, and Tie2 genes in normal ovary with corpus luteum and in ovarian cancer. Oncology. 2002;62(4):340–8.](http://paperpile.com/b/RdymOk/vVxZ)

11. [Guo J, Hu Y, Cao Q, Zhang Y, Jia Y, Liu L, et al. GADD45A is essential for granulosa cells differentiation and ovarian reserve in human and mice. J Cell Mol Med. 2025 Sep;29(17):e70820.](http://paperpile.com/b/RdymOk/lrAG)

12. [Tamura M, Sasano H, Suzuki T, Fukaya T, Watanabe T, Aoki H, et al. Immunohistochemical localization of growth hormone receptor in cyclic human ovaries. Hum Reprod. 1994 Dec;9(12):2259–62.](http://paperpile.com/b/RdymOk/9g8v)

13. [Kitami K, Yoshihara M, Koya Y, Sugiyama M, Iyoshi S, Uno K, et al. Microphthalmia-associated transcription factor-dependent melanoma cell adhesion molecule activation promotes peritoneal metastasis of ovarian cancer. Int J Mol Sci. 2020 Dec 21;21(24):9776.](http://paperpile.com/b/RdymOk/VnKi)

14. [Birgersson M, Indukuri R, Lindquist L, Stepanauskaite L, Luo Q, Deng Q, et al. Ovarian ERβ cistrome and transcriptome reveal chromatin interaction with LRH-1. BMC Biol. 2023 Nov 29;21(1):277.](http://paperpile.com/b/RdymOk/bm6N)

15. [Kim SY, Ebbert K, Cordeiro MH, Romero MM, Whelan KA, Suarez AA, et al. Constitutive activation of PI3K in oocyte induces ovarian granulosa cell tumors. Cancer Res. 2016 Jul 1;76(13):3851–61.](http://paperpile.com/b/RdymOk/CCq3)

16. [Koch J, Foekens J, Timmermans M, Fink W, Wirzbach A, Kramer MD, et al. Human VAT-1: a calcium-regulated activation marker of human epithelial cells. Arch Derm Res. 2003 Sep;295(5):203–10.](http://paperpile.com/b/RdymOk/QYgK)

17. [Gerdes J, Lemke H, Baisch H, Wacker HH, Schwab U, Stein H. Cell cycle analysis of a cell proliferation-associated human nuclear antigen defined by the monoclonal antibody Ki-67. J Immunol. 1984 Oct;133(4):1710–5.](http://paperpile.com/b/RdymOk/pCwm)

18. [Nielsen CF, Zhang T, Barisic M, Kalitsis P, Hudson DF. Topoisomerase IIα is essential for maintenance of mitotic chromosome structure. Proc Natl Acad Sci U S A. 2020 Jun 2;117(22):12131–42.](http://paperpile.com/b/RdymOk/RO2W)

19. [Russell DL, Doyle KMH, Ochsner SA, Sandy JD, Richards JS. Processing and localization of ADAMTS-1 and proteolytic cleavage of versican during cumulus matrix expansion and ovulation. J Biol Chem. 2003 Oct 24;278(43):42330–9.](http://paperpile.com/b/RdymOk/B1y6)

20. [Fang L, Yu Y, Zhang R, He J, Sun YP. Amphiregulin mediates hCG-induced StAR expression and progesterone production in human granulosa cells. Sci Rep. 2016 Apr 26;6:24917.](http://paperpile.com/b/RdymOk/bl8J)

21. [Ko C, Gieske MC, Al-Alem L, Hahn Y, Su W, Gong MC, et al. Endothelin-2 in ovarian follicle rupture. Endocrinology. 2006 Apr;147(4):1770–9.](http://paperpile.com/b/RdymOk/bl2l)

22. [Russell DL, Doyle KMH, Gonzales-Robayna I, Pipaon C, Richards JS. Egr-1 induction in rat granulosa cells by follicle-stimulating hormone and luteinizing hormone: combinatorial regulation by transcription factors cyclic adenosine 3’,5'-monophosphate regulatory element binding protein, serum response factor, sp1, and early growth response factor-1. Mol Endocrinol. 2003 Apr;17(4):520–33.](http://paperpile.com/b/RdymOk/mPtP)

23. [Choi H, Roh J. LH-induced transcriptional regulation of Klf4 expression in granulosa cells occurs via the cAMP/PKA pathway and requires a putative Sp1 binding site. Int J Mol Sci. 2020 Oct 6;21(19):7385.](http://paperpile.com/b/RdymOk/aHAN)

24. [Morris ME, Meinsohn MC, Chauvin M, Saatcioglu HD, Kashiwagi A, Sicher NA, et al. A single-cell atlas of the cycling murine ovary. Elife [Internet]. 2022 Oct 7 [cited 2025 Oct 22];11(e77239). Available from:](http://paperpile.com/b/RdymOk/G93D) <http://dx.doi.org/10.7554/eLife.77239>

25. [Wang S, Zheng Y, Li J, Yu Y, Zhang W, Song M, et al. Single-cell transcriptomic atlas of primate ovarian aging. Cell. 2020 Feb 6;180(3):585–600.e19.](http://paperpile.com/b/RdymOk/t7ca)

26. [Lim H, Paria BC, Das SK, Dinchuk JE, Langenbach R, Trzaskos JM, et al. Multiple female reproductive failures in cyclooxygenase 2-deficient mice. Cell. 1997 Oct 17;91(2):197–208.](http://paperpile.com/b/RdymOk/9ejV)

27. [Park ES, Lind AK, Dahm-Kähler P, Brännström M, Carletti MZ, Christenson LK, et al. RUNX2 transcription factor regulates gene expression in luteinizing granulosa cells of rat ovaries. Mol Endocrinol. 2010 Apr;24(4):846–58.](http://paperpile.com/b/RdymOk/uthA)

28. [Jorgez CJ, Klysik M, Jamin SP, Behringer RR, Matzuk MM. Granulosa cell-specific inactivation of follistatin causes female fertility defects. Mol Endocrinol. 2004 Apr;18(4):953–67.](http://paperpile.com/b/RdymOk/gUIu)

29. [Myers M, Middlebrook BS, Matzuk MM, Pangas SA. Loss of inhibin alpha uncouples oocyte-granulosa cell dynamics and disrupts postnatal folliculogenesis. Dev Biol. 2009 Oct 15;334(2):458–67.](http://paperpile.com/b/RdymOk/4V5q)

30. [M’baye M, Hua G, Khan HA, Yang L. RNAi-mediated knockdown of INHBB increases apoptosis and inhibits steroidogenesis in mouse granulosa cells. J Reprod Dev. 2015 Jun 9;61(5):391–7.](http://paperpile.com/b/RdymOk/oLIF)

31. [Joyce IM, Pendola FL, Wigglesworth K, Eppig JJ. Oocyte regulation of kit ligand expression in mouse ovarian follicles. Dev Biol. 1999 Oct 15;214(2):342–53.](http://paperpile.com/b/RdymOk/7gU8)

32. [Ambrosini G, Adida C, Altieri DC. A novel anti-apoptosis gene, survivin, expressed in cancer and lymphoma. Nat Med. 1997 Aug;3(8):917–21.](http://paperpile.com/b/RdymOk/dTgH)

33. [Sherley JL, Kelly TJ. Regulation of human thymidine kinase during the cell cycle. J Biol Chem. 1988 Jun 15;263(17):8350–8.](http://paperpile.com/b/RdymOk/i1nG)

34. [Townsley FM, Aristarkhov A, Beck S, Hershko A, Ruderman JV. Dominant-negative cyclin-selective ubiquitin carrier protein E2-C/UbcH10 blocks cells in metaphase. Proc Natl Acad Sci U S A. 1997 Mar 18;94(6):2362–7.](http://paperpile.com/b/RdymOk/j7YY)

35. [Han X, Wang R, Zhou Y, Fei L, Sun H, Lai S, et al. Mapping the mouse cell atlas by microwell-seq. Cell. 2018 May 17;173(5):1307.](http://paperpile.com/b/RdymOk/BXbq)

36. [Brayman M, Thathiah A, Carson DD. MUC1: a multifunctional cell surface component of reproductive tissue epithelia. Reprod Biol Endocrinol. 2004 Jan 7;2(1):4.](http://paperpile.com/b/RdymOk/2LIP)

37. [Sim GK, Yagüe J, Nelson J, Marrack P, Palmer E, Augustin A, et al. Primary structure of human T-cell receptor alpha-chain. Nature. 1984;312(5996):771–5.](http://paperpile.com/b/RdymOk/mct3)

38. [Muhl L, Genové G, Leptidis S, Liu J, He L, Mocci G, et al. Single-cell analysis uncovers fibroblast heterogeneity and criteria for fibroblast and mural cell identification and discrimination. Nat Commun. 2020 Aug 7;11(1):3953.](http://paperpile.com/b/RdymOk/1n21)

39. [Sasano H, Mori T, Sasano N, Nagura H, Mason JI. Immunolocalization of 3 beta-hydroxysteroid dehydrogenase in human ovary. J Reprod Fertil. 1990 Jul;89(2):743–51.](http://paperpile.com/b/RdymOk/dxuf)

40. [Satchell L, Glister C, Bleach EC, Glencross RG, Bicknell AB, Dai Y, et al. Ovarian expression of insulin-like peptide 3 (INSL3) and its receptor (RXFP2) during development of bovine antral follicles and corpora lutea and measurement of circulating INSL3 levels during synchronized estrous cycles. Endocrinology. 2013 May;154(5):1897–906.](http://paperpile.com/b/RdymOk/xl3C)

**Table captions:**

**Supplementary Table S2. LC-MS/MS data acquisition parameters for ovarian proteome profiling.** This table summarizes the liquid chromatography and Orbitrap Eclipse Tribrid mass spectrometer settings used for global proteomic analysis of ovaries from 14-month-old mice stimulated with PMSG or PMSG + hCG, as described in the *Mass Spectrometry* section of the Methods. Parameters include, but are not limited to, column configuration, gradient duration and solvent composition, flow rate, scan range, resolution, data-dependent acquisition settings, collision energy, dynamic exclusion, and injection scheme. These settings underlie the peptide identifications reported in Supplementary Table S4 and S7 and Supplementary Figure S3.

**Supplementary Table S3. Pfam domain annotations for Pfam-bearing isoforms included in domain and proteogenomic analyses.** This table lists Pfam-annotated isoforms used in the protein domain analyses (Figure 5) and proteogenomic overlap quantification. For each transcript, the table reports the internal transcript identifier (transcript_id), Pfam accession (pfam_id), Pfam short name (target_name), and Pfam domain description (description). These Pfam-bearing isoforms form the basis for the splice-type-specific domain distributions in Figure 5C and for the set of isoforms cross-referenced to LC-MS/MS detections in Supplementary Figure S3 and Supplementary Table S4.

**Supplementary Table S4. Strict filtered peptide-level LC-MS/MS identifications and mappings to Pfam-bearing isoforms**. This table contains the Perseus-filtered peptide list derived from the MaxQuant peptides.txt output for ovarian LC-MS/MS experiments, after removal of reverse hits, potential contaminants, peptides lacking MS/MS support, and peptides without valid intensities in ≥2 of 3 replicates in at least one treatment group, and after filtering for peptides unique to a single FASTA entry as described in the Methods. Each row corresponds to a peptide and includes: label-free intensities across the six LC-MS/MS runs (Intensity 1A-1C: PMSG; Intensity 2A-2C: PMSG + hCG), uniqueness flags (“Unique (Groups)” and “Unique (Proteins)”), amino-acid composition counts, peptide length, number of missed cleavages, calculated mass, start and end positions, posterior error probability (PEP), Andromeda score, experiment identifiers, peptide and MS/MS IDs, N- and C-terminal cleavage windows, flanking amino acids, associated protein(s) and leading razor protein, taxonomy information, peptide sequence, charge state(s), protein group IDs, modified peptide IDs, evidence IDs, MS/MS IDs, and oxidation (M) site IDs. These data support the proteogenomic overlap analysis that quantifies strict unique-peptide evidence for Pfam-bearing, age-biased isoforms (Figure 5C and Supplementary Figure S3).

**Supplementary Table S5. Cell type- and age-specific isoform abundances for 177 targets validated by paired-end scRNA-seq.** This table reports isoform-level abundances from the orthogonal paired-end 210/90 single-cell RNA-sequencing experiment used to validate long-read splicing events. For each of the 177 targeted isoforms, the table summarizes isoform usage across ovarian cell types and age groups (young vs. aged), derived from alignments of 210-nt R1 reads to the promoter-group isoform FASTA and isoform ratio calculations per (gene, promoter, cell type, age), as described in the Tail uniqueness, minimap alignment, and Scanpy pipeline and Paired-end single-cell RNA-sequencing isoform usage validation sections. These data underlie the cell type-resolved validation of age-biased isoforms in Figure 5 and Supplementary Figure S4, including *Ndufs4*.

**Supplementary Table S6. Ubiquitously detected genes underlying Supplementary Figure S2 B - C and Reactome enrichment.** This Excel file provides the underlying gene lists responsible for the continuous high “fraction of cells expressing” signal (yellow band) observed across ovarian cell types in Supplementary Fig. S2B (PMSG) and S2C (PMSG + hCG), along with Reactome pathway over-representation results for these broadly detected genes. Genes were classified as “ubiquitously detected” if they were detected in ≥ 80% of cells (binary detection) within the relevant scRNA-seq dataset. Supplementary Figure S2 B - C visualizes the fraction of cells expressing genes whose transcripts show significant age-associated splicing changes after PMSG or PMSG + hCG stimulation.

Worksheets:

• Summary: counts of ubiquitous (≥80% detected) genes for S2B and S2C and brief

notes.

• S2B_PMSG_yellowGenes: gene symbols meeting the ≥80% detection criterion in the

S2B (PMSG) gene set, plus a coarse functional category label.

• S2C_PMSGhCG_yellowGenes: same as above for S2C (PMSG + hCG).

• Overlap: union of S2B/S2C ubiquitous genes with boolean indicators (in_S2B_PMSG,

in_S2C_PMSG_hCG) and the functional category.

• S2B_PMSG_TopReactome / S2C_PMSGhCG_TopReactome: top enriched Reactome

terms for each ubiquitous gene set.

• S2B_PMSG_AllReactome / S2C_PMSGhCG_AllReactome: full Reactome enrichment

outputs for each ubiquitous gene set.

• README: brief description of criteria and column definitions.

Reactome column definitions (Reactome sheets): stId (Reactome stable identifier), name (pathway name), hits (number of query genes overlapping the pathway), size (number of genes in the pathway), p (raw enrichment p-value), fdr (multiple-testing–adjusted FDR), –log10(p) (transformed p-value).

**Supplementary Table S7. MaxQuant peptide identifications from proteomics search against translated long-read isoforms.** This Excel file contains the MaxQuant “peptides.txt” output from a database search performed, using a custom protein FASTA derived from translated long-read RNA-seq isoforms. The file reports peptide-level identifications and quantification across proteomics samples corresponding to Sample 1 = PMSG ovaries and Sample 2 = PMSG + hCG ovaries, with three replicates each (A - C). Only the peptides which passed the initial filtering steps (described in the methods) are included in the table. To apply the strict filtering criteria, peptides which were unique to one sequence in the FASTA file can be selected by filtering the “Proteins (Unique)” column for “yes”. These data support the proteogenomic overlap analysis that quantifies strict unique-peptide detection and relaxed evidence for Pfam-bearing, age-biased isoforms (Figure 5C and Supplementary Figure S3).

Key columns (high-level guide):

• Peptide identity/sequence context: *Sequence*, N- and C-terminal cleavage windows,

flanking amino acids.

• Composition/features: amino-acid counts, peptide *Length*, *Missed cleavages*, *Mass*.

• Protein assignment: *Proteins*, *Leading razor protein*, *Start position*, *End position*,

uniqueness fields.

• Confidence metrics: *PEP* (posterior error probability), *Score*, *MS/MS Count*, related IDs.
